# The Evaluation of an Interprofessional QI Program: A Qualitative Study

**DOI:** 10.3390/ijerph191610087

**Published:** 2022-08-15

**Authors:** Ilja M. Brugman, Annelies Visser, Jolanda M. Maaskant, Suzanne E. Geerlings, Anne M. Eskes

**Affiliations:** 1 Department of Surgery, Amsterdam UMC, University of Amsterdam, 1105 AZ Amsterdam, The Netherlands; 2 Emma Children’s Hospital, Amsterdam UMC, University of Amsterdam, 1105 AZ Amsterdam, The Netherlands; 3 Department of Epidemiology and Data Science, Amsterdam UMC, University of Amsterdam, 1105 AZ Amsterdam, The Netherlands; 4 Department of Internal Medicine, Infection, Immunity and Infectious Diseases, Amsterdam UMC, University of Amsterdam, 1105 AZ Amsterdam, The Netherlands; 5 Amsterdam Public Health Research Institute, 1105 AZ Amsterdam, The Netherlands; 6 ACHIEVE, Centre of Applied Research, Faculty of Health, Amsterdam University of Applied Sciences, 1105 BD Amsterdam, The Netherlands

**Keywords:** quality improvement, EBQI, interprofessional education, evaluation, qualitative research, leadership

## Abstract

**Background**: Quality Improvement (QI) is the key for every healthcare organization. QI programs may help healthcare professionals to develop the needed skills for interprofessional collaboration through interprofessional education. Furthermore, the role of diversity in QI teams is not yet fully understood. This evaluation study aimed to obtain in-depth insights into the expectations and experiences of different stakeholders of a hospital-wide interprofessional QI program. **Methods**: This qualitative study builds upon 20 semi-structured interviews with participants and two focus groups with the coaches and program advisory board members of this QI program. Data were coded and analyzed using thematic analysis. **Results**: Three themes emerged from the analysis: “interprofessional education”, “networking” and “motivation: presence with pitfalls”. Working within interprofessional project groups was valuable, because participants with different experiences and skills helped to move the QI project forward. It was simultaneously challenging because IPE was new and revealed problems with hierarchy, communication and planning. Networking was also deemed valuable, but a shared space to keep in contact after finalizing the program was missing. The participants were highly motivated to finish their QI project, but they underestimated the challenges. **Conclusions**: A hospital-wide QI program must explicitly pay attention to interprofessional collaboration and networking. Leaders of the QI program must cherish the motivation of the participants and make sure that the QI projects are realistic.

## 1. Introduction

Quality Improvement (QI) is the key for every healthcare organization, in order to improve safety, effectiveness and patient experiences [1]. The growing attention to QI results from the increasing complexity of patient care, due to the aging populations with more comorbid chronic diseases, and the increase in critically ill patients, which is partly a result of technological developments [2,3]. In addition, patients are more empowered [3]. Responding to these developments, QI relates to both the needs of the patients and the ability of an organization to fulfil these needs, as well as the collaboration and coordination between healthcare professionals [4,5].

Research has shown that interprofessional collaboration (IPC) can improve the quality of patient care, e.g., it improves patient safety and health outcomes [5,6,7,8]. In addition, IPC can positively influence the success of QI programs [9,10,11,12]. One way to foster IPC is by engaging frontline staff in QI projects; this is important as it helps to sustain the improvements [13,14,15]. For example, nurses are of great value in QI programs because they are directly involved in patient care, and, therefore, have in-depth insight into the strengths and weaknesses of hospital systems [15]. However, in order to optimize IPC, skills for collaboration need to be developed and practiced [3,5,16,17]. For this reason, it is important to invest in interprofessional education (IPE) [5,9,12,18]. Given the current developments in healthcare and the benefits of IPC, QI should incorporate IPE [11,12].

There are many different QI programs within healthcare organizations [19,20]. Wong, Levinson and Shojania [20] made a distinction between different organizational frameworks: (1) formal curricula that teach concepts or methods intended to facilitate trainees’ participation in QI activities; (2) educational activities that impart specific related skills; (3) QI initiatives that involve trainees as active or passive participants. The program in this evaluation falls into the first category. Despite the many initiatives, the successful delivery of QI programs should not be taken for granted, as the results of different QI programs vary [9,10]. This can be due to various facilitators and barriers on different levels, such as the organization, the team or the individual [9,21,22]. These contextual factors, such as work environment, should be taken into account when setting up QI projects and should be continuously evaluated in existing QI initiatives [22,23]. Furthermore, according to Rowland et al. [24], more attention should be paid to understanding the role of diversity in QI teams. Therefore, we executed a qualitative evaluation study with the aim of obtaining in-depth insights into the expectations and experiences of different stakeholders (participants, coaches and members of the advisory board) of a hospital-wide interprofessional QI program.

## 2. Materials and Methods

### 2.1. The Program: Quality Improvement Academy

In 2019, we developed and implemented a project-based QI program in Amsterdam University Medical Center (Amsterdam UMC): the Quality Improvement Academy (https://www.qiacademy.nl/, accessed on 10 August 2022) (QI Academy). Amsterdam UMC is a tertiary referral hospital with two locations in the Netherlands. The hospital has more than 16,000 employees and is one of the largest Dutch hospitals. The Amsterdam UMC, as the first hospital in the Netherlands, created the QI academy in collaboration with the Institute of Health Improvement (IHI). The QI Academy aims to educate an interprofessional group of healthcare professionals, who are directly involved in patient care, to plan and carry out QI projects in their local healthcare settings. Participants are offered new skills, support from coaches and a network of colleagues who all work in the field of QI. The program is designed as a 12-month guided experiential learning program and comprises the following three aspects:

1. E-learnings via the IHI Open School [25]. Candidates of the QI Academy individually follow 13 Open School courses, and by finishing all of these courses they earn the Basic Certificate in Quality and Safety.
2. Monthly meetings with a keynote speaker. Speakers are invited based on their experience in Quality and Safety from different perspectives (e.g., professors in Quality and Safety, members of the executive board of hospitals, patients).
3. Project-based learning. The participants work in interprofessional groups to carry out their own QI projects. They receive support from coaches who are skilled and experienced in QI (i.a., cyclical improvement).

Since the first pilot in 2019, the program has expanded step by step, and in 2022 the QI Academy was opened for all healthcare professionals in both locations of the Amsterdam UMC. Over the course of these 3 years, the QI Academy had 73 participants (the first year saw 16 participants, 32 in the second year and 25 in the third year). Of those 73 participants, 54 finished the program (74%) and 19 dropped out (26%) during the course of the program. The maximum capacity of our program is 50 participants per year. The final program is set yearly by the board of the QI Academy which in turn is advised by an advisory board. Over the years, a QI network has been created with the participants, the coaches, and the board members. The first curriculum was evaluated by means of a questionnaire which was filled out at the end of each monthly meeting. Participants were satisfied with the program (average of 8,2 on a scale 1–10). However, this number gained insufficient information to improve the program.

### 2.2. Study Design and Setting

We used a qualitative study design to evaluate the QI program. Interviews were held with participants, and focus groups were organized to involve the coaches and advisory board of the program. The collected data were analyzed using thematic analysis. We considered thematic analysis the appropriate method for answering our research question, as it focuses on shared experiences and meanings among the participants [26]. The study is reported according to the Standards for Reporting Qualitative Research (SRQR) [27].

### 2.3. Participants and Method of Approach

All healthcare professionals who participated in the QI Academy were eligible to participate in the present study. Purposive sampling [28] was used to maximize variability in terms of gender, work experience and professional background in order to capture a wide range of perspectives. New participants were invited for an interview until data saturation was reached. Towards the end of 2021, we invited potential participants by e-mail. In total, 20 participants agreed to participate in the interviews, and 13 participants joined the focus group discussions.

We chose to conduct interviews with the participants to create a safe environment to speak freely, and to be able to obtain a more in-depth insight into their personal experiences. Additionally, coaches and members of the advisory board were invited to participate in the focus groups. Here, we chose focus groups to encourage discussion between the participants. During the period in which the interviews were conducted, different COVID-19 restrictions were applicable within the hospital, which led to most interviews (n = 13) and both focus groups taking place online. The other interviews (n = 7) took place in a quiet room at the hospital at a time of mutual convenience. Apart from the participants and the researchers, no one else was present during the (focus group) interviews to put the participants at ease and enable them to speak freely.

Semi-structured (focus group) interview schedules were developed based on the Context Input Process Product (CIPP) model [29,30], the curriculum and input of the interdisciplinary research team (see Table S1). The trustworthiness was established by the sampling approach, and by generating a non-judgmental atmosphere during the interview [31]. All (focus group) interviews were audio-recorded and transcribed verbatim. The transcription was carried out by an independent organization (Amberscript Global B.V. the Netherlands). They automatically generated the transcripts and checked these transcripts manually to ensure the quality. The researcher (IB) carried out a final check on the provided transcripts.

### 2.4. The Role of the Researchers

The interviewer (IB) had no formal hierarchical relationship with the participants. She had a master’s degree in Applied Cultural and Social Anthropology and had previous experience with interviewing. All (focus group) interviews were conducted by the same researcher (IB). The research team consisted of a nurse scientist, an anthropologist, a clinical epidemiologist, an internal medicine specialist/professor in quality of care, and a staff advisor quality and innovation, who were all actively involved in the QI Academy.

### 2.5. Validation

During the (focus group) interviews field notes were taken such as striking topics or emotions. These notes were used to help the interpretation of data and credibility of the findings. The first interview was a pilot interview to test the interview procedure and interview schedule. No major changes were made. Therefore, the results were included in the data. The interview participants received transcripts of the interview and the focus group participants were given a summary of the focus group discussion. They were asked for comments or corrections if necessary (member check) [31]. No substantive changes were requested.

### 2.6. Data Analysis

All data were analysed using the thematic analysis approach described by Braun and Clark [32]. This approach consists of the following steps: (1) (re)reading all transcripts to become familiar with the data; (2) generating initial codes; (3) searching for themes; (4) reviewing the themes; (5) defining and naming themes; (6) producing the final report.

The first three interviews were coded by two researchers, independently (IMB and LR). The results were compared and discussed, and no major differences were found in the coding and interpretation of the data. Therefore, the remaining interviews and focus groups were analysed by the first author (IMB). Field notes were re-read to contextualize and check the coding. This investigator triangulation deepened the researchers’ understanding of the experiences and expectations of the participants and increased the credibility of results [33]. After completing the initial coding (step 1–2), the research team sorted the emerged codes into main themes during several meetings (step 3–5). Data management was carried out using MAXQDA Plus 2022 (release 22.0.1).

## 3. Results

### 3.1. Sample Description

The interviews took place between October 2021 and January 2022 with an average duration of 30 min. We invited 37 participants for an interview, of whom 17 declined. The 20 participants interviewed represented 27% of the total number of participants of the QI Academy from the start. The two focus groups took place in November 2021, and took approximately 60 min. The advisory board was represented by seven out of the 13 invited members in one focus group. During the focus group for the coaches, six out of the 11 invited coaches were present. An overview of the characteristics of the participants is presented in Table 1.

### 3.2. Thematic Analysis

**Figure 1:**
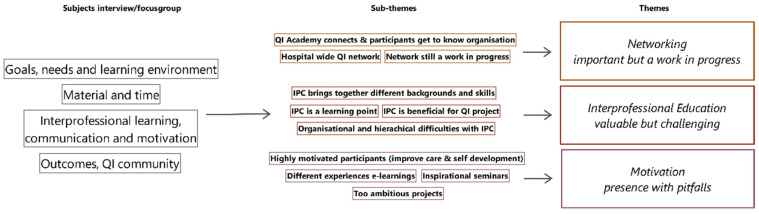
The three themes that emerged from the analysis.

Three themes emerged from the analysis of both the interviews and focus group discussions (see Figure 1): “interprofessional education”, “networking” and “motivation: presence with pitfalls”. The themes are described below, illustrated with quotations of the participants.

#### 3.2.1. Interprofessional Education

The interprofessional feature of the QI Academy appeared to be an important reason for the participants to enroll in the program. Most of the participants managed to create an interprofessional project group. Working on a project within an interprofessional group was often new to them. During the QI projects the participants became even more aware of the benefits of interprofessional collaboration. The participants’ various experiences, insights and knowledge of the hospital, originating from different professional backgrounds, were considered important for the success of the projects.

*“[When working with an interprofessional group] I think your circle of influence is bigger”*.

(Physician, interview 10)

*“No doubt [this collaboration was of added value]. Because [the physician-assistants] have different insights, connections and other moments of contact”*.

(Senior nurse, interview 13)

In addition, several participants experienced working in interdisciplinary groups as an opportunity to learn how to work together, and, thus, as beneficial for their daily practice. The advisory board also considered this to be an important learning point.

*“So I find it useful to practice that a bit, how do you do that? How can you work together in a team in a way it is pleasant for me and it also has added value for the other”*.

(Physician, interview 3)

*“That you learn how you can tackle things together”*.

(Senior nurse, interview 8)

*“I think collaborating is also very important, that they learn how to do that”*.

(Advisory board member A, focus group advisory board)

Participants became aware that the different functions and professional backgrounds brought different skills together in the project groups. For example, doctors seemed to work more theoretically or analytically, while nurses were used to performing more practical work. The coaches also noticed this while mentoring the different project groups. The participants and coaches emphasized that both theoretical and practical skills are valuable for a QI project.

*“[Because improvement often is a multidisciplinary process] you have to work together. So, yes I would pledge to keep [the program] diverse because I also think they can learn from each other”*.

(Coach B, focus group coaches)

*“Because there are cultural differences between physicians and nurses. They have a different way of looking at things. [You have a] broader view when you work together with different disciplines within one group”*.

(Team leader, interview 2)

*“If the nurses on the ward have a completely different view, which was the case, then it is really useful to be able to complement each other”*.

(Physician, interview 17)

Apart from the described benefits of interprofessional education, the participants also experienced difficulties. Several coaches noticed that creating an interprofessional project group was not always easy. This finding is supported by the fact that out of the 20 interviewees, 14 participated in an interprofessional project group, while six of them did not. Some participants also felt like the QI Academy program forced them to choose between an interesting project or an interprofessional group.

*“When you want to make an improvement, everybody- the supervisors, would like to see an improvement that can be implemented on your own ward. […] Or we could join a group, which was mixed, but where you would not have affinity [with the project]. Or we formed a group with two nurses […] and it would be a project that was really useful”*.

(Senior nurse, Interview 4)

During the execution of the projects, participants acknowledged that it was sometimes difficult to step beyond the hierarchical positions and to work together as equals. Moreover, differences in the amount of experience in QI and project management hampered collaboration. In addition, participants mentioned difficulties in the organization and continuity of the project, because of different working schedules.

*“Working interdisciplinary, because it is easy to say, but, to my surprise, it can be such a slow process. There is so much hesitation to work within a multidisciplinary group”*.

(Coach A, focus group coaches)

*“A project together with physicians and nurses where everybody has a different schedule, and without getting dedicated time, is just hard [to organize]”*.

(Senior nurse, interview 13)

#### 3.2.2. Networking

The value of networking emerged in nearly all the interviews. The participants mentioned that the QI Academy gave them the opportunity to connect with colleagues whom they would not have met otherwise. Several participants explained that they became engaged in a network of people interested in QI during their participation in the program. This network increased the possibility to learn from each other and broadened their scope.

*“It is really nice to get to know new people. It is educational when group members are both involved in direct patient care and from supportive disciplines; they can learn so much from each other”*.

(Nurse, interview 11)

*“The network [is important] as I mentioned earlier. I noticed how a hospital operates, you start working within disciplines really quick and you forget to think outside of this framework”*.

(Team leader, interview 5)

*“I think it is, for young physicians and nurses and many other people within the organization, an accessible opportunity to be involved in interdisciplinary matters that can have a significant impact on healthcare”*.

(Physician, interview 20)

Members of the advisory board also mentioned that they expanded their QI network within the hospital. They emphasized the importance of this hospital-wide network in which so many people of different professional backgrounds, all dedicated to Quality Improvement, can meet.

*“What is also valuable is that I now know who participated at the different wards. So when I’m walking around, I’m like, oh, but I know you”*.

(Advisory board member B, focus group advisory board)

*“I do believe that quality and safety should not be underestimated. […] And that has resulted in the fact that you now get more and more people, who at least have that realization. […] Of course it has to slowly spread through the organization”*.

(Advisory board member C, focus group advisory board)

Although the Quality Improvement network is expanding, the advisory board recognized it is still work in progress. This is illustrated by one of the coaches, who mentioned that contact with participants of the project groups ended abruptly after the curriculum. Indeed, not all interviewees considered themselves engaged in the QI Academy network. Thus, an alumni program is considered important, but it is not yet in place, even though it is deemed important for the success of the QI Academy.

*“Maybe there could be some kind of follow-up […] because suddenly it was over. And I don’t know how the last period was for my students [the participants], which I find a pity”*.

(Coach C, focus group coaches)

*“So you definitely have to build a knowledge infrastructure around [the program], where you actually continue to build on the knowledge that all those people need. That has to be nourished”*.

(Advisory board member D, focus group advisory board)

#### 3.2.3. Motivation: Presence with Pitfalls

The participants reported different experiences with the IHI e-learnings. Some learned a lot, while others felt the need to seek additional information. Most participants found the e-learnings American-centered and, therefore, not always applicable in the Dutch context. Others found them difficult because they required analytic thinking and some struggled with the English language.

*“[The e-learnings] mostly consisted of clicking through extremely American examples. […] Luckily I found some things interesting enough to [myself] add some depth”*.

(Physician, interview 19)

*“Each time I thought: oh no, I have to do the e-learnings again. […] But after finishing I thought: well this was really interesting.”*

(Senior nurse, interview 8)

*“It was a combination of relevance and the English language. My English is quite good but I noticed I struggled a bit now and then. […] But it was mainly the relevance. Some [e-learnings] I really liked, but others I found less interesting.”*

(Team leader, interview 2)

The participants of the QI Academy expressed enthusiasm about QI and showed an intrinsic motivation to make a sustainable change with their projects. The participants were highly focused on a successful result of the projects. In addition, they valued the possibility of self-development.

*“[I want to] develop myself and deepen my knowledge and skills, in order to improve the quality of care for the patients. But also to make it applicable to the ward and really implement [the results], and get the team on board of the change”*.

(Paramedic, interview 9)

*“People participate voluntarily. […] So it really comes from the people themselves. I think that is really unique”*.

(Member of advisory board B, focus group advisory board)

*“Yes, I think it is really good that […] as soon as you see something, you don’t start to fiddle around, but [you know you can] change things and you know things can change”*.

(Physician–researcher, interview 14)

The monthly lectures by experts were highly motivational and educational for the participants.

*“Several seminars, there were some really good ones that were inspiring. They make you think about what you are doing, what quality is, and why some things do not work”*.

(Medical specialist, interview 16)

*“Mostly the webinars and the e-learnings, each time I kept looking forward to what am I going to learn this time? What will they talk about? And also the improvement project, you have some motivation, like I really want to finish this. […] And I want things to be well organized for the patient”*.

(Team leader, interview 2)

The participants also mentioned challenges, such as formulating realistic expectations and time management. In the focus group with the coaches, it became clear that the high motivation of the participants could also be a pitfall. Due to their enthusiasm ambitious projects were started, but sometimes ended with feelings of disappointment because they appeared too complicated or too much work.

*“There is an enormous ‘aha enlightening’ among all those students [while carrying out their projects using the PDSA cycle], they think: oh, so that’s how it works. And half-way they realize and think: wow, that it would be this much work I wouldn’t have thought!”*

(Coach C, focus group coaches)

## 4. Discussion

We executed a qualitative evaluation study with the aim to obtain in-depth insights into the expectations and experiences of different stakeholders of a hospital-wide interprofessional QI program. Based on interviews and focus groups with the participants, coaches and members of the advisory board of the program, three themes emerged: ‘Interprofessional education’, ‘Networking’ and ‘Motivation: presence with pitfalls’.

All the stakeholders interviewed mentioned that IPE is valuable and essential for QI, but also challenging. While working on the QI projects it became clear to the participants and coaches that having different complementary experiences and skills helped to move the project forward. Learning how to collaborate (interprofessional) was often one of the goals of the participants, as working in interprofessional teams was considered relevant in the hospital care setting. These insights are congruent with previous literature, as healthcare professionals are expected to have the skills needed for interdisciplinary collaboration and continuously working on quality of care by adapting everyday practices to new insights [3,16,17]. IPE in QI programs can be one way to learn these skills [18,34].

In our study, we also found that IPE comes with its own challenges. The stakeholders endorsed these challenges in the process of creating and working in interprofessional QI projects. In the design and implementation of these projects, they experienced dealing with hierarchy, different levels of experience, and organizational challenges. These challenges are described in the existing literature as known barriers for IPC, e.g., insufficient time [35,36]; hierarchy [35,36]; professional socialization and working within silos [35,37,38]; and poor communication, including a lack of understanding of roles and responsibilities [35,36]. Nurses in particular seem to struggle with hierarchical factors and territorial concerns [15,37,39], issues of time management [15] and a lack of self-confidence and (in)formal support [15,40].

The literature also shows that these challenges are worth overcoming. There are promising results about combining QI and IPE [11,12]. For most of the interviewed participants in our study, working in interprofessional project groups was new and often even a learning point. It is suggested that if participants are more experienced in IPE, collaboration will become less challenging [5,34]. Therefore, the different healthcare professionals should be introduced to IPE at the start of their career, preferably as early as during their education [3,5,34,37]. It may be valuable to bring more explicit attention to working and learning inter-professionally within a QI program by discussing topics such as professional socialization and the accompanying culture and power differences [35,38,41]. In addition, the different opinions concerning QI, the consequences for the shared project, and the different expectations and roles might deserve more attention [35,38].

The participants, coaches and advisory board members see the importance of creating a network for QI. However, most of the participants and coaches have not been in contact with each other since finalizing the program. Moreover, the advisory board recognizes that an alumni network is still a work in progress. It is recognized in the literature that networks give the opportunity to build bridges between disciplines, going beyond traditional hierarchical structures, which can help to meet long-term goals [42]. Human relations are believed to play a key role in QI [43]. In order to enhance a quality culture and continuously improve the quality of care, the QI Academy should focus on networking, communication, and coalition building [43]. One way to create a shared space for the program participants to share knowledge and network is to make use of virtual communities of practice. They have a few advantages for interprofessional groups as they can overcome both the geographical and time barriers of the time-pressured healthcare workers [44]. Furthermore, creating a shared space helps to overcome social and professional boundaries within the hospital [44]. Next to creating a shared space, ongoing staff and leadership engagement are crucial for sustainable QI projects [14,45]. Support from managers and having dedicated time for QI are two ways to ensure this [13,45].

While looking at the motivations, it became clear that the participants were highly motivated. What stands out is their focus on making a change and improving quality of care. This high motivation could also have been a pitfall when participants started QI projects that were too ambitious. Therefore, it is important to start with realistic and manageable QI projects and to set clear expectations with the participants [46,47]. The participants were most enthusiast about the seminars, which shows the importance of inspirational leaders in this program [43].

### Limitations

This study focusses specifically on the evaluation of a QI program embedded in one hospital in the Netherlands only, and the results must be seen within this context. Secondly, although we tried to obtain a representative group of participants, not all of the individuals we invited participated, which may have created bias. For example, the participants who did not have a strong connection with or opinion about the QI Academy may have been left out. In addition, we could not compare the characteristics of the interviewees with the total group of participants involved in the QI Academy. As for the focus groups, we could not compare the characteristics with the total group, but we did have an attendance of over 50%.

The sample was limited to 20 interviewees and 13 participants in the focus groups. Although more participants might have brought in more information, we experienced data saturation at the end of the data collection period. Moreover, for the thematic analyses, a minimum of 10 interviews is considered sufficient [48].

## 5. Conclusions

The qualitative evaluation of a hospital-wide QI program provided in-depth information on the expectations and experiences of the different stakeholders. The QI program is considered important to improve the quality of care, with special attention to interprofessional education and networking. The exploration of the experienced challenges are valuable, as they give direction to future improvements of the QI program.

Interprofessional collaboration can become even more educational and successful if there is more explicit attention paid to different expectations and roles during the program. Therefore, this should be added to the curriculum. In order for the program to achieve more long-term effects and build an associated network there should be more attention for networking, communication, and coalition building. Finally, keeping the QI-projects small will help to keep the participants motivated.

## Figures and Tables

**Table 1:** Interview participants characteristics.

**Interviews (n)**	
Total	20
**Interviews per cohort (n)**	
2019–2020	6
2020–2021	7
2021–2022	7
**Gender (n)**	
Male	7
Female	13
**Work experience (years)**	
Mean	11.9
Range	1–32
**Profession (n)**	
Administrative function	1
Medical professional (in training)	2
(Senior) nurse	6
Paramedic	2
Physician (in training)	6
Physician–researcher)	1
Team leader	2
Completion program * (n)	
Finished	17
Dropped out	3

* up to July 2022.

## Data Availability

The data presented in this study are not publicly available due to the small scale of this study but are available upon reasonable request from the corresponding author.

## Supplementary Materials

The following are available online at https://www.mdpi.com/article/10.3390/ijerph191610087/s1, Table S1: Interview schedules according to CIPP method.
