# Supplementary material for: The Evaluation of an Interprofessional QI Program: A Qualitative Study
Source: Int J Environ Res Public Health. 2022 Aug 15;19(16):10087. doi: 10.3390/ijerph191610087 (PMC9408409; doi:10.3390/ijerph191610087)
Supplement: Supplementary file 1 [file ijerph-19-10087-s001.zip › ijerph-1819646-supplementary.pdf]

International Journal of
Environmental Research and Public Health

MDPI

Supplementary Materials

Table S1. Interview schedules according to CIPP-method.

		Material for Analysis
**Context**	*Goal(s)*	Curriculum<br>Focus groups<br>Interviews
	Needs/ Requirements	Focus groups<br>Interviews
	Educational environment: History of the Amsterdam UMC, background of quality improvement in healthcare	Focus groups<br>Literature QI
**Input**	*Course material, time*	Curriculum<br>Focus groups<br>Interviews
	Human resources: fellow participants, coaches, colleagues in Amsterdam UMC, QI network, advisory board	Focus groups<br>Interviews
**Process**	*Interdisciplinary learning*	Focus groups<br>Interviews
	Communication	Focus groups<br>Interviews
	Motivation participants	Focus groups<br>Interviews
**Product**	*Goals reached?*	Curriculum<br>Focus groups<br>Interviews
	Outcomes: Knowledge gained (in practice) & Result QI project	Focus groups<br>Interviews
	Creating a community of quality experts within Amsterdam UMC	Focus groups<br>Interviews
